# Update: Influenza Activity — United States, October 1–November 25, 2017

**DOI:** 10.15585/mmwr.mm6648a2

**Published:** 2017-12-08

**Authors:** Vivien G. Dugan, Lenee Blanton, Anwar Isa Abd Elal, Noreen Alabi, John Barnes, Lynnette Brammer, Erin Burns, Charisse N. Cummings, Todd Davis, Brendan Flannery, Alicia M. Fry, Shikha Garg, Rebecca Garten, Larisa Gubareva, Yunho Jang, Krista Kniss, Natalie Kramer, Stephen Lindstrom, Desiree Mustaquim, Alissa O’Halloran, Sonja J. Olsen, Wendy Sessions, Calli Taylor, Susan Trock, Xiyan Xu, David E. Wentworth, Jacqueline Katz, Daniel Jernigan

**Affiliations:** 1 Influenza Division, National Center for Immunization and Respiratory Diseases, CDC.

Influenza activity in the United States was low during October 2017, but has been increasing since the beginning of November. Influenza A viruses have been most commonly identified, with influenza A(H3N2) viruses predominating. Several influenza activity indicators were higher than is typically seen for this time of year. The majority of influenza viruses characterized during this period were genetically or antigenically similar to the 2017–18 Northern Hemisphere cell-grown vaccine reference viruses. These data indicate that currently circulating viruses have not undergone significant antigenic drift; however, circulating A(H3N2) viruses are antigenically less similar to egg-grown A(H3N2) viruses used for producing the majority of influenza vaccines in the United States. It is difficult to predict which influenza viruses will predominate in the 2017–18 influenza season; however, in recent past seasons in which A(H3N2) viruses predominated, hospitalizations and deaths were more common, and the effectiveness of the vaccine was lower. Annual influenza vaccination is recommended for all persons aged ≥6 months who do not have contraindications. Multiple influenza vaccines are approved and recommended for use during the 2017–18 season, and vaccination should continue to be offered as long as influenza viruses are circulating and unexpired vaccine is available. This report summarizes U.S. influenza activity* during October 1–November 25, 2017 (surveillance weeks 40–47).^†^

## Viral Surveillance

**FIGURE 1:**
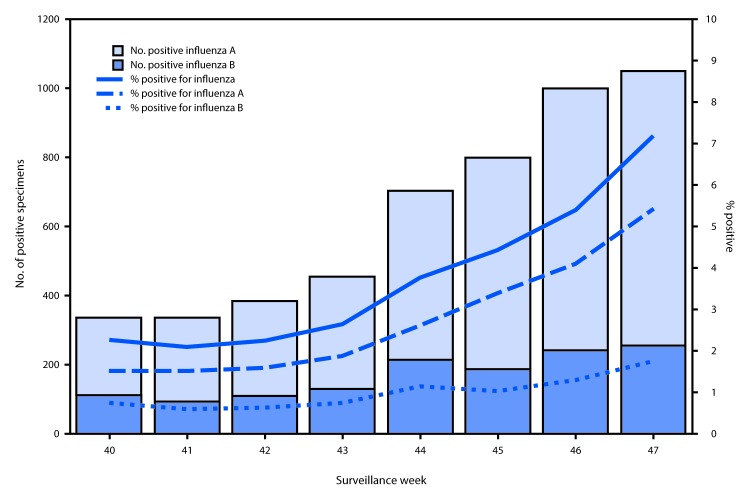
Number* and percentage of respiratory specimens testing positive for influenza reported by clinical laboratories, by influenza virus type and surveillance week — United States, October 1–November 25, 2017^†^ * Specimens from 5,070 (3.7%) of 135,202 persons tested positive during October 1–November 25, 2017. ^†^ As of December 1, 2017.

U.S. World Health Organization (WHO) and National Respiratory and Enteric Virus Surveillance System laboratories, which include both public health and clinical laboratories throughout the United States, contribute to virologic surveillance for influenza. During October 1–November 25, 2017, clinical laboratories tested 135,202 specimens for influenza virus; 5,070 (3.7%) specimens tested positive for influenza virus (Figure 1), including 3,723 (73.4%) that tested positive for influenza A viruses and 1,347 (26.6%) that tested positive for influenza B viruses.

**FIGURE 2:**
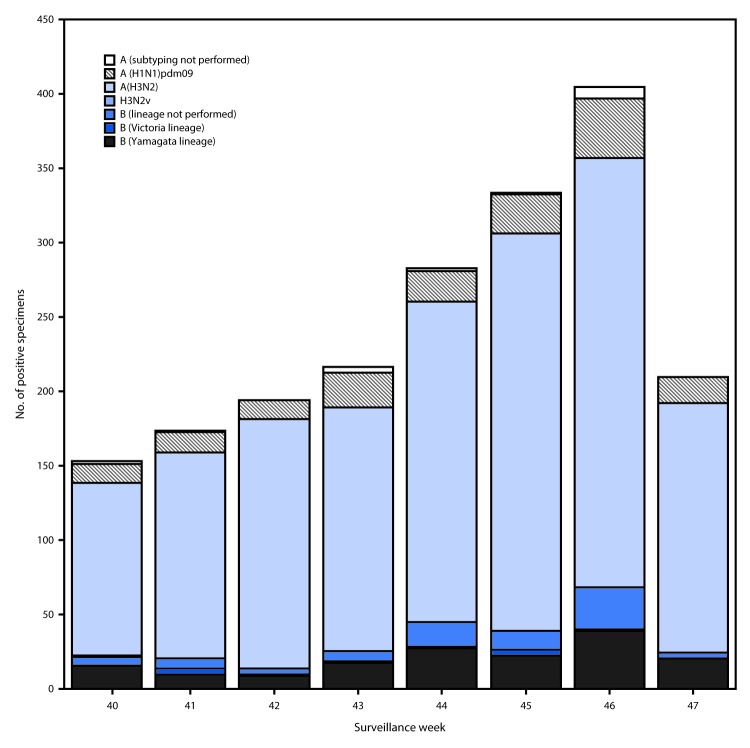
Number* of respiratory specimens testing positive for influenza reported by public health laboratories, by influenza virus type, subtype/lineage, and surveillance week — United States, October 1–November 25, 2017^†^ * N = 1,970. ^†^ As of December 1, 2017.

Public health laboratories tested 8,777 specimens during October 1–November 25, 2017, and 1,969 (22.4%) were positive for influenza, including 1,714 (87%) influenza A and 255 (13%) influenza B viruses (Figure 2). Among the 1,696 influenza A viruses subtyped, 1,527 (90%) were influenza A(H3N2) viruses, and 169 (10%) were influenza A(H1N1)pdm09 viruses. Influenza B virus lineage information was available for 170 (66.1%) tested influenza B viruses; 159 (93.5%) belonged to the B/Yamagata lineage and 11 (6.5%) to the B/Victoria lineage.

Data on age were available for 1,737 influenza-positive patients tested by public health laboratories. Overall, 163 (9.4%) persons were aged 0–4 years, 475 (27.3%) were aged 5–24 years, 576 (33.2%) were aged 25–64 years, and 523 (30.1%) were aged ≥65 years. Influenza A(H3N2) viruses were predominant among all age groups, accounting for 69.9% of viruses identified among persons aged 0–4 years and 87.8% of viruses reported among persons aged ≥65 years. The largest proportion of reported influenza B viruses occurred in persons aged 5–24 years; influenza B viruses accounted for 16.6% of the viruses reported for that age group.

## Novel Influenza A Viruses

Five human infections with novel influenza A viruses were reported to CDC by five states (one each in Colorado, Iowa, Michigan, Nebraska, and Ohio) during October 1–November 25, 2017. All of these were variant^§^ virus infections (human infections with influenza viruses that normally circulate in swine). Two infections were caused by influenza A(H3N2)v viruses, two by influenza A(H1N2)v viruses, and one by an influenza A(H1N1)v virus. The patient from Colorado reported exposure to swine at an agricultural event during the week preceding illness onset. The patient from Iowa had direct contact with swine during the week preceding illness onset. The patient from Michigan was a close contact of a person with laboratory-confirmed A(H3N2)v virus infection that had been reported earlier this year. Although that patient also reported exposure to swine, it occurred more than a week before illness onset, which is outside the typical incubation period. It is possible that this infection resulted from limited human-to-human transmission. The patient from Nebraska reported no contact with swine during the week preceding illness onset; however, a household member did report exposure to swine. The patient from Ohio reported exposure to swine at an agricultural fair during the week preceding illness onset. Two of the five patients were children aged <18 years, one patient was an adult aged 18–44 years, and two patients were adults aged ≥45 years. Two of the patients were hospitalized, and all have fully recovered from their illness. No ongoing human-to-human transmission was identified.

The A(H3N2)v viruses detected in Michigan and Nebraska had a hemagglutinin (HA) gene segment derived from a seasonal human H3N2 virus that was likely introduced into swine by reverse zoonosis (i.e., humans infecting swine) in 2010. These viruses were closely related to H3N2 viruses known to circulate in the U.S. swine population (*1*), as well as to variant virus infections detected in Delaware, Maryland, Michigan, North Dakota, Ohio, and Pennsylvania during May–September 2017 (*2*). The A(H1N2)v viruses detected in Colorado and Ohio had HA gene segments from the delta sublineage of the classical swine H1 HA lineage (*3*). The HA and neuraminidase (NA) gene segments of this virus were closely related to 2016/2017 H1N2 influenza viruses known to circulate in the U.S. swine population and have been sporadically detected in other A(H1N2)v virus infections. The A(H1N1)v virus detected in Iowa had HA and NA gene segments derived from the seasonal human H1N1pdm09 virus that was likely introduced into swine by a recent reverse zoonosis. This virus was closely related to H1N1 influenza viruses currently circulating in the U.S. swine population.

## Antigenic and Genetic Characterization of Influenza Viruses

In the United States, public health laboratories participating in influenza surveillance as WHO collaborating laboratories are asked to submit a subset of influenza-positive respiratory specimens to CDC for virus characterization according to specific guidelines (*4*). CDC characterizes influenza viruses through one or more laboratory tests, including genomic sequencing, antigenic characterization by hemagglutination inhibition (HI), or neutralization assays. Circulating viruses that have been isolated and propagated in mammalian cell culture are evaluated for antigenic similarity with cell culture–propagated reference viruses representing the recommended vaccine components of the Northern Hemisphere 2017–18 vaccine (*5*). This process establishes whether antigenic drift from the vaccine reference viruses has occurred.

All influenza-positive surveillance specimens submitted for surveillance and received by CDC are sequenced by next generation sequencing (NGS), using previously described genomic enrichment practices (*6*–*8*) adapted by CDC. The genomic data from the NGS pipeline are analyzed to determine the genetic identity of circulating viruses and submitted to public databases (GenBank or GISAID EpiFlu). Data obtained from antigenic characterization are important in the assessment of the similarity between reference vaccine viruses and circulating viruses. In vitro antigenic characterization data generated through HI assays or virus neutralization assays are used to assess whether genetic changes in circulating viruses affect antigenicity, which subsequently might affect vaccine effectiveness.

Since the 2014–15 season, many influenza A(H3N2) viruses lack sufficient hemagglutination titers for antigenic characterization using HI assays. Therefore, a subset of influenza A(H3N2) viruses are selected for antigenic characterization using the virus neutralization focus reduction assay to assess the ability of various antisera to neutralize infectivity of the test viruses. CDC has antigenically or genetically characterized 277 influenza viruses collected and submitted by U.S. laboratories since October 1, 2017, including 38 influenza A(H1N1)pdm09 viruses, 187 influenza A(H3N2) viruses, and 52 influenza B viruses.

**FIGURE 3:**
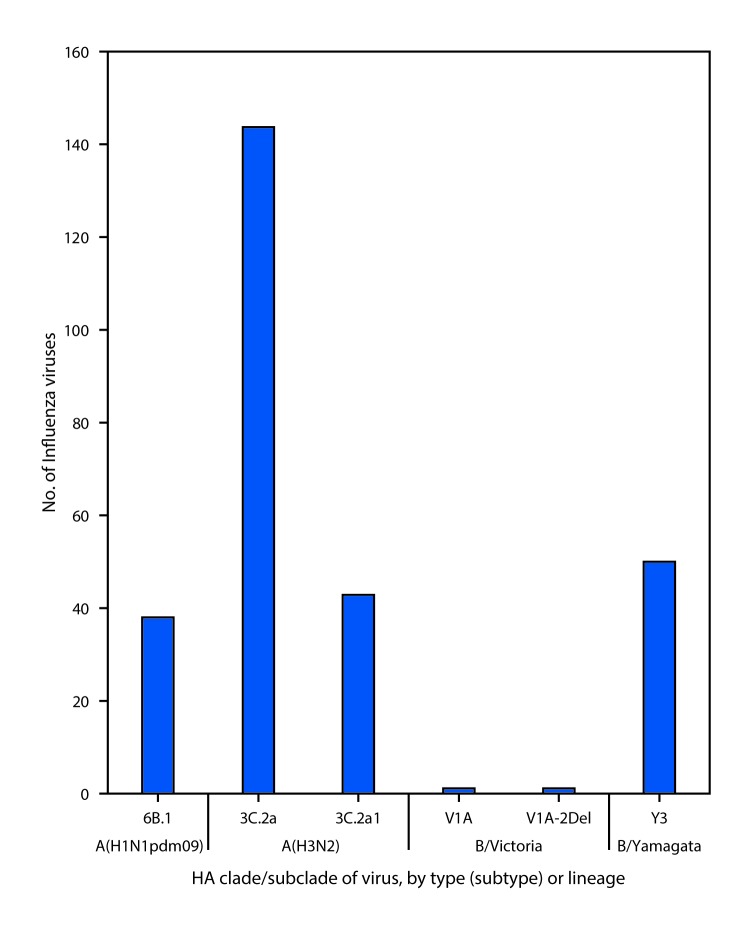
Genetic characterization of U.S. viruses collected during October 1, 2017–November 25, 2017* **Abbreviation:** HA = hemagglutinin. * As of December 1, 2017.

Phylogenetic analysis of the HA gene segments from 38 A(H1N1)pdm09 viruses collected since October 1, 2017, showed that all belonged to subclade 6B.1 (Figure 3). Thirteen A(H1N1)pdm09 viruses were analyzed using HI assays with ferret antisera, and all of these viruses were antigenically similar to the cell culture–propagated 6B.1 virus A/Michigan/45/2015, the reference virus representing the A(H1N1)pdm09 vaccine virus for the 2017–18 Northern Hemisphere influenza season.

One hundred and eighty-seven influenza A(H3N2) viruses collected since October 1, 2017, were sequenced, and phylogenetic analysis of the HA gene segments illustrated that multiple clades/subclades were cocirculating (Figure 3). The HA gene segments belonged to clade 3C.2a or subclade 3C.2a1, with 3C.2a predominating (Figure 3). Sixty-four influenza A(H3N2) viruses were antigenically characterized, and 63 (98.4%) were well-inhibited (reacting at titers that were within fourfold of the homologous virus titer) by ferret antisera raised against A/Michigan/15/2014 (3C.2a), a cell-propagated A/Hong Kong/4801/2014–like reference virus representing the A(H3N2) component of the 2017–18 Northern Hemisphere influenza vaccines. Although considerable genetic diversity has been observed among H3N2 viruses, there has been no evidence of significant antigenic drift in the limited number of H3N2 viruses tested from this season. A smaller number, 45 (70.3%) of viruses tested, were well-inhibited by antiserum raised against egg-propagated A/Hong Kong/4801/2014 reference virus representing the A(H3N2) vaccine component. This is likely because of egg-adaptive amino acid changes in the HA of the egg-propagated virus.

Two influenza B/Victoria-lineage viruses were sequenced and phylogenetically analyzed, and the HA gene segment of both viruses belonged to genetic clade V1A, the same genetic clade as the vaccine reference virus, B/Brisbane/60/2008. However, the HA gene segment of one virus has a 6-nucleotide deletion (encoding amino acids 162 and 163) and viruses like this, abbreviated as V1A-2Del, were previously reported (*2*). This V1A-2Del virus was poorly inhibited (reacting at titers that were eightfold or more reduced compared with the homologous virus titer) with antisera raised to cell culture–propagated B/Brisbane/60/2008, the reference virus representing the B/Victoria lineage component of 2017–18 Northern Hemisphere vaccines.

Phylogenetic analysis of 50 influenza B/Yamagata-lineage viruses show that the HA gene segments belonged to clade Y3 (Figure 3). Fourteen B/Yamagata lineage viruses were antigenically characterized, and all were antigenically similar to the cell culture–propagated B/Phuket/3073/2013, the reference virus representing the B/Yamagata-lineage component of quadrivalent vaccines for the 2017–18 Northern Hemisphere influenza season.

## Antiviral Resistance of Influenza Viruses

The WHO Collaborating Center for Surveillance, Epidemiology, and Control of Influenza at CDC tested 291 influenza virus specimens (41 influenza A(H1N1)pdm09, 200 influenza A(H3N2), and 50 influenza B viruses) collected in the United States since October 1, 2017, for resistance to the influenza NA inhibitor antiviral medications oseltamivir, zanamivir, and peramivir, drugs currently approved for use against seasonal influenza. All 291 influenza viruses tested were sensitive to all three antiviral medications. High levels of resistance to the adamantanes (amantadine and rimantadine) persist among influenza A(H1N1)pdm09 and influenza A(H3N2) viruses. Adamantane drugs are not recommended for use against influenza at this time.

## Outpatient Illness Surveillance

**FIGURE 4:**
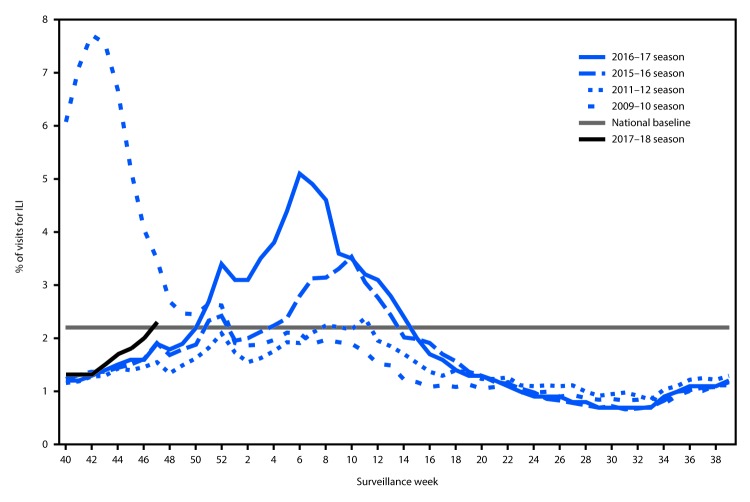
Percentage of outpatient visits for influenza-like illness (ILI)* reported to CDC, by surveillance week — U.S. Outpatient Influenza-Like Illness Surveillance Network, 2017–18 influenza season and selected previous influenza seasons^†^ * Defined as fever (temperature of ≥100°F [≥37.8°C], oral or equivalent) and cough or sore throat, without a known cause other than influenza. ^†^ As of December 1, 2017.

During October 1–November 25, 2017, the weekly percentage of outpatient visits for influenza-like illness^¶^ (ILI) to heath care providers participating in the U.S. Outpatient Influenza-like Illness Surveillance Network (ILINet) ranged from 1.3% to 2.3%. During the week ending November 25, 2.3% of patient visits reported through ILINet were for ILI, which is above the national baseline** level of 2.2% (Figure 4). The increase in the percentage of patient visits for ILI during the week ending November 25 (surveillance week 47) might be influenced in part by a reduction in routine health care visits during the holidays, as has occurred in previous seasons. During the week ending November 25, four of 10 U.S. Department of Health and Human Services regions^††^ (Regions 1, 4, 6, and 7) reported a percentage of outpatient visits for ILI at or above their region-specific baseline levels.

ILINet data are used to produce a weekly jurisdiction-level measure of ILI activity,^§§^ ranging from minimal to high. For the week ending November 25, three states (Louisiana, Mississippi, and South Carolina) experienced high ILI activity; one state (Georgia) experienced moderate ILI activity; 10 states (Alabama, Alaska, Arizona, Hawaii, Massachusetts, Nebraska, Oklahoma, South Dakota, Texas, and Virginia) experienced low ILI activity; the District of Columbia, New York City, and 36 states (Arkansas, California, Colorado, Connecticut, Delaware, Florida, Idaho, Illinois, Indiana, Iowa, Kansas, Kentucky, Maine, Maryland, Michigan, Minnesota, Missouri, Montana, Nevada, New Hampshire, New Jersey, New Mexico, New York, North Carolina, North Dakota, Ohio, Oregon, Pennsylvania, Rhode Island, Tennessee, Utah, Vermont, Washington, West Virginia, Wisconsin, and Wyoming) experienced minimal ILI activity; and Puerto Rico had insufficient data to calculate an ILI activity level.

## Geographic Spread of Influenza Activity

Influenza activity levels reported by state and territorial epidemiologists indicate the geographic spread of influenza viruses. For the week ending November 25 (surveillance week 47), four states (Georgia, Louisiana, Massachusetts, and Oklahoma) reported widespread activity.^¶¶^ Guam and 10 states (Arkansas, Connecticut, Kentucky, Maine, Mississippi, New Hampshire, North Dakota, Oregon, South Carolina, and Washington) reported regional activity. Puerto Rico and 24 states (Alabama, Alaska, Arizona, California, Colorado, Florida, Hawaii, Illinois, Kansas, Maryland, Minnesota, Missouri, Nebraska, New Jersey, New Mexico, New York, Ohio, Pennsylvania, South Dakota, Tennessee, Texas, Utah, Wisconsin, and Wyoming) reported local activity. The District of Columbia, the U.S. Virgin Islands, and 12 states (Delaware, Idaho, Indiana, Iowa, Michigan, Montana, Nevada, North Carolina, Rhode Island, Vermont, Virginia, and West Virginia) reported sporadic activity.

## Influenza-Associated Hospitalizations

CDC monitors hospitalizations associated with laboratory-confirmed influenza infections in adults and children through the Influenza Hospitalization Surveillance Network (FluSurv-NET),*** which covers approximately 27 million persons (9% of the U.S. population). During October 1, 2017–November 25, 2017, a total of 566 laboratory-confirmed influenza-related hospitalizations were reported, with a cumulative incidence for all age groups of 2.0 per 100,000 population. The hospitalization rate was highest among persons aged ≥65 years, who accounted for approximately 50% of reported influenza-associated hospitalizations.

The cumulative influenza hospitalization rates per 100,000 population during October 1, 2017–November 25, 2017, for persons aged 0–4 years, 5–17 years, 18–49 years, 50–64 years, and ≥65 years were 1.6, 0.6, 0.8, 2.4, and 7.3, respectively. Among all hospitalizations, 484 (85.5%) were associated with influenza A virus infections, 80 (14.1%) with influenza B virus infections, and two (0.4%) with influenza A virus and influenza B virus coinfections. Among the 146 patients for whom influenza A subtype information was available, 127 (87.0%) were infected with influenza A(H3N2) viruses, and 19 (13.0%) were infected with influenza A(H1N1)pdm09 viruses.

## Pneumonia and Influenza-Associated Mortality

CDC tracks pneumonia and influenza (P&I)–attributed deaths through the National Center for Health Statistics (NCHS) Mortality Reporting System. The percentages of deaths attributed to P&I are released 2 weeks after the week of death to allow for collection of sufficient data to produce a stable P&I mortality percentage. Based on data from NCHS available on November 30, 2017, 5.7% of all U.S. deaths occurring during the week ending November 11, 2017, (surveillance week 45) were attributed to P&I. This percentage is below the epidemic threshold^†††^ of 6.5% for week 45. Since October 1, the weekly percentage of deaths attributed to P&I has ranged from 5.7% to 6.2% and has not exceeded the epidemic threshold for this season. P&I percentages for recent weeks might be artificially low because of a backlog of records requiring manual processing, and the percentage of deaths caused by P&I is higher among manually coded death certificates than among machine-coded death certificates. The percentage of deaths caused by P&I will likely increase as more data become available.

## Influenza-Associated Pediatric Mortality

As of November 25, 2017 (surveillance week 47), five laboratory-confirmed influenza-associated pediatric deaths occurring during the 2017–18 season were reported to CDC. Two deaths were associated with an influenza A(H1N1)pdm09 virus infection, two were associated with an influenza A(H3) virus infection, and one was associated with an influenza A virus for which no subtyping was performed. Since influenza-associated pediatric mortality became a nationally notifiable condition in 2004, the number of influenza-associated pediatric deaths per season has ranged from 37 to 171, excluding the 2009 pandemic, when 358 pediatric deaths were reported to CDC during April 15, 2009–October 2, 2010.

## Discussion

Influenza activity in the United States for the 2017–18 season was low during October but has been increasing since early November. The timing of influenza activity often varies; however, peak influenza activity in the United States most commonly occurs during December–February, and substantial influenza activity can be observed through May. It is difficult to predict when influenza activity will peak for the current season; however, influenza activity will increase in the coming weeks. During October 1–November 25, 2017, A(H3N2) viruses were most commonly reported, but A(H1N1)pdm09 and influenza B viruses also were reported. The majority of influenza viruses collected in the United States since October 1, 2017, were characterized antigenically or genetically as being similar to the cell-grown reference viruses representing the 2017–18 Northern Hemisphere influenza vaccine viruses, indicating that significant antigenic drift has not occurred at this time. However, some currently circulating A(H3N2) viruses are less similar to egg-adapted viruses used for production of the majority of U.S. influenza vaccines.

Although influenza vaccine effectiveness can range widely from season to season, influenza vaccination is the most effective currently available method to prevent influenza and its complications. However, less than half of the U.S. population has been vaccinated in recent influenza seasons. Even with influenza vaccine effectiveness in the range of 30% to 60%, influenza vaccination prevents millions of infections and medical visits and tens of thousands of influenza-associated hospitalizations each year in the United States.^§§§^ Health care providers should recommend influenza vaccine now and throughout the influenza season to all unvaccinated persons aged ≥6 months who do not have contraindications. Children aged 6 months–8 years who had not previously received a total of ≥2 doses of any trivalent or quadrivalent influenza vaccine (doses do not have to be received in the same influenza season) before July 1, 2017, require 2 doses for the 2017–18 season. The interval between the 2 doses should be at least 4 weeks (*5*).

For the 2017–18 season, manufacturers projected they would supply the United States with 151 million–166 million doses of injectable influenza vaccine. As of November 24, 2017, approximately 148.2 million doses of vaccine had already been distributed. Influenza vaccination coverage estimates for this season show coverage similar to the same time last season among the general population. Survey data collected through early November 2017 indicate that 38.6% of all persons aged ≥6 months reported receiving flu vaccination (compared with 39.8% at this time last season). This leaves approximately 3 out of 5 persons in the United States unprotected against influenza. These estimates are reported on the CDC website (https://www.cdc.gov/flu/fluvaxview/). 

The majority of influenza viruses collected this season, although small in number, have been antigenically and genetically characterized as being similar to the cell-grown reference viruses representing the 2017–18 Northern Hemisphere influenza vaccine viruses. The lack of significant antigenic drift observed for recently circulating influenza viruses further suggests that vaccination with the Northern Hemisphere influenza vaccine should offer similar protection as past seasons when cell-grown reference vaccine viruses were most similar to circulating viruses. Vaccine effectiveness can vary between influenza seasons and by virus type or subtype. Studies have shown reduced vaccine effectiveness against A(H3N2) viruses (30–40%), in the absence of significant antigenic drift, when compared with A(H1N1) and influenza B viruses (*9*). This reduction in effectiveness might result, in part, from the egg propagation of influenza A(H3N2) vaccine virus components required for most influenza vaccine products licensed in the United States. For example, egg adaptation of current A(H3N2) viruses typically results in a loss of N-linked glycosylation motif at residues 158-160 of the HA protein, which is within an important antibody epitope (site B). Other factors that might also contribute to the reduced effectiveness against A(H3N2) viruses include the naturally occurring, high level of genetic diversity and rapid evolutionary rate of this particular subtype and modification of the immune response to vaccine because of prior infection or vaccination. Vaccine effectiveness studies are needed to ascertain the level of protection that influenza vaccination provides to the population, but these data will not be available until later in the season.

Influenza antiviral medications are an important adjunct to vaccination in the treatment and prevention of influenza. Treatment with influenza antiviral medications as close to the onset of illness as possible is recommended for patients with confirmed or suspected influenza who have severe, complicated, or progressive illness; who require hospitalization; or who are at high risk for influenza complications. Antiviral treatment should be initiated as soon as possible for patients who are at high risk for complications or who are severely ill with suspected influenza infection, even if rapid antigen-detection influenza diagnostic test results are negative (*10*).

Influenza surveillance reports for the United States are posted online weekly (https://www.cdc.gov/flu/weekly). Additional information regarding influenza viruses, influenza surveillance, influenza vaccine, influenza antiviral medications, and novel influenza A infections in humans is available online (https://www.cdc.gov/flu).

**Summary**

**What is already known about this topic?**

CDC collects, compiles, and analyzes data on influenza activity year-round in the United States. Timing of influenza activity and predominant circulating influenza viruses varies by season.

**What is added by this report?**

Influenza activity remained low in the United States during October 2017, but has been increasing since November. As of November 25, influenza A(H3N2) viruses were the most commonly identified viruses. The majority of influenza viruses collected in the United States since October 1, 2017, were characterized antigenically or genetically as being similar to the cell-grown reference viruses representing the 2017–18 Northern Hemisphere influenza vaccine viruses. All influenza viruses tested to date have been sensitive to the antiviral drugs oseltamivir, zanamivir, and peramivir.

**What are the implications for public health practice?**

In the United States, annual influenza vaccination can reduce the likelihood of becoming ill with influenza and transmitting the virus to others and is recommended for all persons aged ≥6 months. Annual influenza vaccination offers optimal protection regardless of whether the vaccine composition has changed since the previous season. Although vaccination is the best method for preventing and reducing the impact of influenza, antiviral medications are an important adjunct. Early treatment with influenza antiviral medications is recommended for patients with confirmed or suspected influenza (either seasonal influenza or novel influenza virus infection) who have severe, complicated, or progressive illness; who require hospitalization; or who are at high risk for influenza-related complications.

## Footnotes

* The CDC influenza surveillance system collects five categories of information from eight data sources: 1) viral surveillance (U.S. World Health Organization collaborating laboratories, the National Respiratory and Enteric Virus Surveillance System, and novel influenza A virus case reporting); 2) outpatient illness surveillance (U.S. Outpatient Influenza-Like Illness Surveillance Network); 3) mortality (National Center for Health Statistics Mortality Surveillance System and influenza-associated pediatric mortality reports); 4) hospitalizations (FluSurv-NET, which includes the Emerging Infections Program and surveillance in three additional states); and 5) summary of the geographic spread of influenza (state and territorial epidemiologist reports). https://www.cdc.gov/flu/weekly/fluactivitysurv.htm.

† Data as of December 1, 2017.

§ Influenza viruses that circulate in swine are called swine influenza viruses when isolated from swine but are called variant influenza viruses when isolated from humans. Seasonal influenza viruses that circulate worldwide in the human population have important antigenic and genetic differences from influenza viruses circulating in swine.

¶ Defined as a fever (temperature ≥100°F [≥37.8°C], oral or equivalent) and cough or sore throat, without a known cause other than influenza.

** The national and regional baselines are the mean percentage of visits for ILI during noninfluenza weeks for the previous three seasons plus two standard deviations. Noninfluenza weeks are defined as periods of ≥2 consecutive weeks in which each week accounted for <2% of the season’s total number of specimens that tested positive for influenza in public health laboratories. National and regional percentages of patient visits for ILI are weighted based on state population. Use of the national baseline for regional data is not appropriate.

†† The 10 regions include the following jurisdictions. *Region 1:* Connecticut, Maine, Massachusetts, New Hampshire, Rhode Island, and Vermont; *Region 2:* New Jersey, New York, Puerto Rico, and the U.S. Virgin Islands; *Region 3:* Delaware, District of Columbia, Maryland, Pennsylvania, Virginia, and West Virginia; *Region 4:* Alabama, Florida, Georgia, Kentucky, Mississippi, North Carolina, South Carolina, and Tennessee; *Region 5:* Illinois, Indiana, Michigan, Minnesota, Ohio, and Wisconsin; *Region 6:* Arkansas, Louisiana, New Mexico, Oklahoma, and Texas; *Region 7:* Iowa, Kansas, Missouri, and Nebraska; *Region 8:* Colorado, Montana, North Dakota, South Dakota, Utah, and Wyoming; *Region 9:* Arizona, California, Hawaii, Nevada, American Samoa, Commonwealth of the Northern Mariana Islands, Federated States of Micronesia, Guam, Marshall Islands, and Republic of Palau; *Region 10:* Alaska, Idaho, Oregon, and Washington.

§§ Activity levels are based on the percentage of outpatient visits in a jurisdiction attributed to ILI and are compared with the average percentage of ILI visits that occur during weeks with little or no influenza virus circulation. Activity levels range from minimal, corresponding to ILI activity from outpatient clinics at or below the average, to high, corresponding to ILI activity from outpatient clinics much higher than the average. Because the clinical definition of ILI is nonspecific, not all ILI is caused by influenza; however, when combined with laboratory data, the information on ILI activity provides a clearer picture of influenza activity in the United States.

¶¶ Levels of activity are 1) no activity; 2) sporadic: isolated laboratory-confirmed influenza cases or a laboratory-confirmed outbreak in one institution, with no increase in activity; 3) local: increased ILI, or two or more institutional outbreaks (ILI or laboratory-confirmed influenza) in one region of the state, with recent laboratory evidence of influenza in that region; virus activity no greater than sporadic in other regions; 4) regional: increased ILI activity or institutional outbreaks (ILI or laboratory-confirmed influenza) in two or more outbreaks, but less than half of the regions in the state with recent laboratory evidence of influenza in those regions; and 5) widespread: increased ILI activity or institutional outbreaks (ILI or laboratory-confirmed influenza) in at least half the regions in the state, with recent laboratory evidence of influenza in the state.

*** FluSurv-NET conducts population-based surveillance for laboratory-confirmed, influenza-associated hospitalizations in children and adolescents aged <18 years (since the 2003–04 influenza season) and adults aged ≥18 years (since the 2005–06 influenza season). The FluSurv-NET covers approximately 70 counties in the 10 Emerging Infections Program states (California, Colorado, Connecticut, Georgia, Maryland, Minnesota, New Mexico, New York, Oregon, and Tennessee) and additional Influenza Hospitalization Surveillance Project (IHSP) states. IHSP began during the 2009–10 season to enhance surveillance during the 2009 H1N1 pandemic. IHSP sites included Idaho, Iowa, Michigan, Oklahoma, and South Dakota during the 2009–10 season; Idaho, Michigan, Ohio, Oklahoma, Rhode Island, and Utah during the 2010–11 season; Michigan, Ohio, Rhode Island, and Utah during the 2011–12 season; Iowa, Michigan, Ohio, Rhode Island, and Utah during the 2012–13 season; and Michigan, Ohio, and Utah during the 2013–14, 2014–15, 2015–16, 2016–17, and 2017–18 seasons. Cumulative unadjusted incidence rates are calculated using CDC’s National Center for Health Statistics population estimates for the counties included in the surveillance catchment area. Laboratory confirmation is dependent on clinician-ordered influenza testing, and testing for influenza often is underutilized because of the poor reliability of rapid test results and greater reliance on clinical diagnosis for influenza. Therefore, cases identified as part of influenza hospitalization surveillance likely are an underestimation of the actual number of persons hospitalized with influenza.

††† The seasonal baseline proportion of P&I deaths is projected using a robust regression procedure, in which a periodic regression model is applied to the observed percentage of deaths from P&I that were reported by the National Center for Health Statistics Mortality Surveillance System during the preceding 5 years. The epidemic threshold is set at 1.645 standard deviations above the seasonal baseline.

§§§ Estimated influenza illnesses, medical visits, hospitalizations, and deaths averted by vaccination in the United States. https://www.cdc.gov/flu/about/disease/2015-16.htm.

## References

[1] Bowman AS, Walia RR, Nolting JM, Influenza A/H3N2 virus in swine at agricultural fairs and transmission to humans, Michigan and Ohio, USA, 2016. Emerg Infect Dis 2017;23:1551–5. 10.3201/eid2309.170847 28820376 PMC5572863

[2] Blanton L, Wentworth DE, Alabi N, Update: influenza activity—United States and worldwide, May 21–September 23, 2017. MMWR Morb Mortal Wkly Rep 2017;66:1043–51. 10.15585/mmwr.mm6639a3 28981486 PMC5720887

[3] Anderson TK, Macken CA, Lewis NS, A phylogeny-based global nomenclature system and automated annotation tool for H1 hemagglutinin genes from swine influenza A viruses. MSphere 2016;1:e00275–16. 10.1128/mSphere.00275-16 27981236 PMC5156671

[4] Association of Public Health Laboratories. Influenza virologic surveillance right size roadmap. 1st ed. Silver Spring, MD: Association of Public Health Laboratories; 2013. https://www.aphl.org/AboutAPHL/publications/Documents/ID_July2013_Influenza-Virologic-Surveillance-Right-Size-Roadmap.pdf

[5] Grohskopf LA, Sokolow LZ, Broder KR, Prevention and control of seasonal influenza with vaccines: recommendations of the Advisory Committee on Immunization Practices—United States, 2017–18 influenza season. MMWR Recomm Rep 2017;66(No. RR-2). 10.15585/mmwr.rr6602a1 28841201 PMC5837399

[6] Zhou B, Donnelly ME, Scholes DT, Single-reaction genomic amplification accelerates sequencing and vaccine production for classical and swine origin human influenza a viruses. J Virol 2009;83:10309–13. 10.1128/JVI.01109-09 19605485 PMC2748056

[7] Zhou B, Wentworth DE. Influenza A virus molecular virology techniques. Methods Mol Biol 2012;865:175–92. 10.1007/978-1-61779-621-0_11 22528160

[8] Zhou B, Lin X, Wang W, Universal influenza B virus genomic amplification facilitates sequencing, diagnostics, and reverse genetics. J Clin Microbiol 2014;52:1330–7. 10.1128/JCM.03265-13 24501036 PMC3993638

[9] Belongia EA, Simpson MD, King JP, Variable influenza vaccine effectiveness by subtype: a systematic review and meta-analysis of test-negative design studies. Lancet Infect Dis 2016;16:942–51. 10.1016/S1473-3099(16)00129-8 27061888

[10] Fiore AE, Fry A, Shay D, Gubareva L, Bresee JS, Uyeki TM. Antiviral agents for the treatment and chemoprophylaxis of influenza—recommendations of the Advisory Committee on Immunization Practices (ACIP). MMWR Recomm Rep 2011;60(No. RR-1). 21248682

